# Safe‐by‐Design: Stakeholders’ Perceptions and Expectations of How to Deal with Uncertain Risks of Emerging Biotechnologies in the Netherlands

**DOI:** 10.1111/risa.13501

**Published:** 2020-05-18

**Authors:** Britte Bouchaut, Lotte Asveld

**Affiliations:** ^1^ Department of Biotechnology Delft University of Technology Delft The Netherlands

**Keywords:** Biotechnology, inherent safety, risk governance, Safe‐by‐Design

## Abstract

Advanced gene editing techniques such as Clustered Regularly Interspaced Short Palindromic Repeat (CRISPR)/Cas have increased the pace of developments in the field of industrial biotechnology. Such techniques imply new possibilities when working with living organisms, possibly leading to uncertain risks. In the Netherlands, current policy fails to address these uncertain risks because risk classification is determined process‐wise (i.e., genetically modified organism [GMO] and non‐GMO), there is a strong focus on quantifiable risks, and the linearity within current governance (science–policy–society) hinders iterative communication between stakeholders, leaving limited room to anticipate uncertainties at an early stage of development. A suggested concept to overcome these shortcomings is the Safe‐by‐Design (SbD) approach, which, theoretically, allows stakeholders to iteratively incorporate safety measures throughout a technology's development process, creating a dynamic environment for the anticipation of uncertain risks. Although this concept originates from chemical engineering and is already widely applied in nanotechnology, for the field of biotechnology, there is no agreed upon definition yet. To explore the possibilities of SbD for future governance of biotechnology, we should gain insight in how various stakeholders perceive notions of risk, safety, and inherent safety, and what this implies for the applicability of SbD for risk governance concerning industrial biotechnology. Our empirical research reveals three main themes: (1) diverging expectations with regard to safety and risks, and to establish an acceptable level of risk; (2) different applications of SbD and inherent safety, namely, product‐ and process‐wise; and (3) unclarity in allocating responsibilities to stakeholders in the development process of a biotechnology and within society.

## 1 INTRODUCTION

Developments in the field of biotechnology have been a topic of discussion since the emergence of genetically modified organisms (GMOs) at the beginning of the 1970s (Berg et al., 1975). Public debate reached its peak during the mid 1990s around the issue of unknown consequences (Hanssen, Dijkstra, Sleenhoff, Frewer, & Gutteling, 2018). Although most debates revolved around applications of agricultural (i.e., green) biotechnology, these discussions also negatively affected the image of industrial (i.e., white) biotechnology. Today, gene editing techniques are causing societal turmoil due to their uncertain risks. In terms of white biotechnology, the application of Clustered Regularly Interspaced Short Palindromic Repeat (CRISPR) technology may offer endless possibilities but could also be accompanied by unforeseen risks, for example, off‐target mutations (Gorter de Vries et al., 2018). Such undesired consequences could negatively affect the public, animals and the environment, and these concerns have reignited the ongoing GMO debate, especially with regard to risk governance applied to all strands of biotechnology, that is, red, green, and white. For the sake of clarity, in this article we use the term *risk governance* to refer to the broad notion of risk‐related decision‐making processes regarding emerging biotechnologies (van Asselt & Renn, 2011).

In the summer of 2018, the Court of Justice of the European Union (ECJ) in Luxembourg, ruled that organisms treated with CRISPR technology should be classified as GMOs (Purnhagen et al., 2018). The main concern arising from this decision is that the focus is too much on quantifiable risks (Callaway, 2018b). This narrow focus offers little flexibility for the further development of CRISPR applications and for dealing with uncertain risks that might accompany this type of biotechnology. As a result, this ruling rekindled the discussion about the development of adequate governance, especially in terms of risk assessment and the proper classification thereof (Callaway, 2018a). Although many have called for measures to shift the focus from quantifiable risks to uncertain risks (Callaway, 2018b; Kupferschmidt, 2018), there is no consensus on the best way to establish adequate risk governance in practice. However, suggestions have been made that adequate risk governance should entail collaboration and co‐development of knowledge between governmental decisionmakers and other stakeholders such as scientists, risk assessors, or other experts in the field (Linkov et al., 2018; Trump et al., 2019). This way, when information regarding quantifiable risks is lacking in early stages of development, quantitative data can be complemented with insights and experimental data from experts (e.g., researchers) in order to gain insight in the balance between the known risks, and possible societal implications (Linkov et al., 2018). However, as there also tends to be a “disciplinary culture” among experts (Ndoh, Cummings, & Kuzma, 2020), a broad inclusion of perspectives would be important to establish appropriate risk governance.

Several policy advisory bodies in the Netherlands have suggested that the concept of Safe‐by‐Design (SbD) could lead us toward the appropriate governance of emerging biotechnologies (Bureau KLB, 2018; Cogem, 2016; Ministerie van Infrastructuur en Waterstaat, 2019; Stemerding & de Vriend, 2018). Theoretically, this approach can include a wide range of stakeholders, establishing co‐development of knowledge to learn about the biotechnologies’ potential impacts. The SbD approach is already applied in the domains of chemical engineering and nanotechnology, and comprises both engineered and procedural safety by “using materials and process conditions that are less hazardous” (Bollinger et al., 1996; Khan & Amyotte, 2003). This refers to the idea of designing specifically for the notion of safety by iteratively integrating knowledge about the adverse effects of materials (van de Poel & Robaey, 2017). Within the domain of biotechnology, the SbD approach is still relatively new and its application should be different from the traditional applications. Yet, there is no concrete definition of the concept for biotechnology, or an explication of how exactly the approach could be applied within this domain. As mentioned, safe biotechnology is a contentious issue on which various stakeholders have different perspectives that need to be teased out in order to arrive at a meaningful use of SbD.

In this article, we pose the question: “How do various stakeholders perceive notions of risk, safety and inherent safety, and what does this imply for the applicability of SbD for risk governance in industrial biotechnology in the Netherlands?” We found that stakeholders hold widely diverging views on the notion of acceptable risk, the allocation of responsibilities, and whether the focus should be on the product or the process, or perhaps both. Because these notions are not aligned, it is hard to reach agreement on what level of risk is acceptable, making it more difficult to apply SbD in an appropriate way. In addition, results illustrate that defining SbD within the context of (white) biotechnology is complex, and would require more research.

## 2 METHODS

### 2.1 Design

This study used a three‐step research approach comprising a literature study, semistructured interviews and a stakeholder workshop1All data (e.g., form of consent, interview protocol, coding protocol, and transcripts) are available upon request via https://doi.org/10.17026/dans-z8a-7p5p.. The first step focused on studies devoted to perceptions of risk and safety in relation to biotechnology, and on the concept of SbD applied in different engineering fields (e.g., chemical engineering, nanotechnology). Studies in the field of biotechnology are mainly associated with perceptions of and attitudes toward GMOs, relevant to the field of white (industrial) and green (food and agriculture) biotechnology. Results from the literature study functioned as input for the interviews.

### 2.2 Interviews

The interviews were carried out in the light of a bigger project, and served the goal of gaining insight in: (1) current policies involving biotechnology and synthetic biology; (2) safety and risks in the development of synthetic biology applications; (3) current interactions between science, policy, and society; and (4) tasks and responsibilities within the overall development process of biotechnologies. For this study, the set of questions was complemented with an extra set of questions specifically focusing on the concept of SbD and perceptions of risks, safety, and inherent safety. Given that the interviewees are working in different domains (i.e., industry, societal sphere, regulatory body, or academia), this helped clarifying how these notions are addressed and used by the interviewees, how these notions relate to the concept of SbD in their perspective, and whether there are differences in these. The interviews followed a semistructured approach that left enough room for interviewees to go into detail when the researchers felt that such was necessary.

Interviews (Ntot = 12) with experts in the field of industrial biotechnology from the Netherlands were conducted in the period May–July 2018 and in February 2019. The interviewees were selected based on their experience (all holding senior positions) and professional domain, namely, industry (ID) (*N* = 2), societal sphere (SO) (*N* = 1), policymaking or regulatory body (PM) (*N* = 4), academia, or independent consultancy (AE) (*N* = 5). At the start of each interview, the interviewee signed a form giving consent to record the interview. After the interview, a transcript was sent to the interviewee for any remarks or corrections. Upon receiving the interviewee's approval, the transcript was coded and analyzed accordingly.

### 2.3 Stakeholder Workshop

The results from the interviews functioned as input for a stakeholder workshop that was held in November 2018 in The Hague, the Netherlands. The aim of the workshop was to clarify recent and future challenges posed by the current regulatory framework for biotechnologies in the Netherlands and to explore the merits of an SbD strategy as a solution to these challenges. The output and preliminary results from the interviews were discussed with all participants and functioned as a reflection on the results obtained so far.

A variety of stakeholders (Ntot = 22) active in the fields of academia (*N* = 7), Dutch governance institutes (*N* = 8), consultancy (*N* = 3), NGOs (*N* = 2), and industry (*N* = 2) participated in the stakeholder workshop, of which most of the interviewees2Nine of the 12 interviewees participated in the stakeholder workshop.. All participants were selected based on their knowledge of and experience in the field of industrial biotechnology in the Netherlands, all holding senior positions in their designated profession, except for one PhD researcher.

## 3 THEORY

### 3.1 Safe‐by‐Design

SbD is an engineering concept for risk management that originated in the field of chemical engineering and is heavily applied in the field of nanotechnology (Kelty, 2009; Schwarz‐Plaschg, Kallhoff, & Eisenberger, 2017; van de Poel & Robaey, 2017). SbD comprises both engineered and procedural safety (Khan & Amyotte, 2003), and is usually referred to as “reducing or eliminating hazards by using materials and process conditions that are less hazardous” (Bollinger et al., 1996; Kahn & Amyotte, 2003). This refers to the idea of designing specifically for the notion of safety by integrating knowledge about the adverse effects of materials (e.g., chemicals or nanomaterials) on human health, animals, and the environment into the design process of a technology (Schwarz‐Plaschg et al., 2017). Literature regarding SbD in the context of chemical engineering or nanotechnology assumes that there is (adequate) knowledge of the used chemicals or nanomaterials (Nau & Scholz, 2019) and that safety can be treated like a property of materials or products. However, the actual usage of such materials in later stages is hereby excluded (Schwarz‐Plaschg et al., 2017). Therefore, for cutting‐edge technologies, such as nanomedicines, it can be hard to adopt SbD principles as these technologies have not reached the same level of maturity as common nanomaterials (Yan et al., 2019). In terms of industrially applied biotechnologies, emerging gene editing techniques such as CRISPR have also not reached the level of matureness to already oversee all (possible) consequences.

Dealing with uncertainties calls for measures different from those used in traditional risk assessment, which addresses and regulates technologies assuming these are fully developed and ready to enter the market (Schwarz‐Plaschg et al., 2017). In that perspective, SbD can be seen as a strategy to shift regulatory and political decisions toward scientists or other engaged stakeholders. Recalling that adequate risk governance should comprise co‐development of knowledge, specifically when data about risks turns out to be insufficient in the early stages of development (Linkov et al., 2018), the concept of SbD enables this by iteratively engaging different stakeholders throughout a biotechnology's development process. When collectively designing with safety in mind, different stakeholders might see different issues arising due to their differing perceptions (Ndoh et al., 2020; Robaey, 2018). However, when many stakeholders are involved in a biotechnology's development process and the focus is on designing for safety, it is important that all the stakeholders’ expectations, notions and perceptions are known and aligned. Any mismatches in notions (feelings of safety and security, sustainability) or expectations (“high” or “low” levels of safety) might lead to difficulties in choosing “the right” design options, making it difficult to reach a collective design with an adequate safety level.

It is currently being explored how the concept of SbD can be applied in technical domains such as biotechnology and synthetic biology. To get a better idea about the suitability of this concept for use in these domains, two types of SbD applications must be distinguished: upstream and downstream (Doorn, Schuurbiers, van de Poel, & Gorman, 2013; Powell, 2007; Schwarz‐Plaschg et al., 2017).

### 3.2 Product Applied SbD

Literature coming from chemical engineering or nanotechnology describes the concept of SbD as safety measures specifically applied *upstream*; aimed at the product itself or the technical components. Examples of these types of measures are the replacement of hazardous chemicals, or adaptation of the process or product synthesis (Kraegeloh, Suarez‐merino, Sluijters, & Micheletti, 2018; Schwarz‐Plaschg et al., 2017; van de Poel & Robaey, 2017). The choice between two chemical compounds having comparable properties but, for example, different levels of toxicity, can be made in a quantifiable way. Regarding safety, the compound having the lower level of toxicity would be preferred in this case. Within this article, we refer to these types of measures with *product‐applied* SbD. Within the field of biotechnology and synthetic biology, measures such as biocontainment (i.e., building in genetic safeguards) are examples of product‐applied SbD applications (Robaey, 2018).

### 3.3 Process‐Applied SbD

In addition to safety measures specifically applied to technical components, there are also measures that are applied *downstream* and might involve decision making at other levels, for example, policy level. Examples of such measures are licensing and monitoring—and in that sense, weighing risks against benefits—, or any other measure that would require the active involvement of multiple stakeholders. Within this article, we refer to such measures with *process‐applied* SbD.

The biggest difference between product‐ and process‐applied SbD lies in the decision‐making process on what is an acceptable level of risk. From a product (upstream) perspective, these decisions are mostly routinely and can be dealt with quantitatively, as it is usually known which risks accompany the usage of certain raw materials or synthesis pathways. From a process (downstream) perspective, the decision regarding what level of risks is acceptable can be more complex, as more uncertainties have to be taken into account. When dealing with new biotechnologies, for example, CRISPR, it is difficult to foresee any future issues or risks due to a lack of experience (van de Poel & Robaey, 2017), complicating the decision‐making process in terms of the “ideal” balance between risks and safety and making it more subjective. In addition, although a certain usage is devised for a biotechnology, in practice, this can turn out differently because different users are involved. In that respect, we can argue that although the norms and values applied to a biotechnology's development process in general will not change, the weight given to them can. For example, safety can be given more weight in the early stages of development, whereas sustainability could become more important at a later stage. And as many stakeholders are involved, reaching a consensus about what weight should be given to which norms and values might be difficult.

### 3.4 Inherent Safety

In literature, strategies and measures for early and iterative safety considerations throughout a technology's development process are frequently referred to as *inherent safety* (Amyotte, Goraya, Hendershot, & Khan, 2007; Kletz, 1996, 2003; Nau & Scholz, 2019; Schwarz‐Plaschg et al., 2017; Yan et al., 2019). In the domains where SbD is already being applied, for example, chemical engineering, the term *inherent* refers to the focus on changing the process at an early stage to eliminate hazards, rather than developing add‐on features to control them (Khan & Amyotte, 2003). In relation to SbD, both notions aim to act upon safety issues by adapting processes during early stages of development to reduce or eliminate potential uncertain risks. However, there is reason to believe that the term *inherent* creates differing expectations and notions among stakeholders, for example in terms of “lower” or “higher” levels of safety, leading to complications with regard to collectively establishing acceptable levels of safety in practice.

Literally translated, *inherent safety* refers to something being intrinsically or built‐in “safe,” hinting at *absolute* safety. The suggestion of something being *absolutely*, namely, 100%, safe is contrary to an engineering point of view, which acknowledges that achieving 100% safety is currently not possible (Khan & Amyotte, 2003; Schmidt, 2008). In addition, it appears that inherent safety has different meanings in different engineering disciplines. Within the traditional engineering disciplines, inherent safety has a rather straightforward definition: “In safety engineering, inherent safety refers to the elimination of hazards, for example, by replacing dangerous substances or processes by less dangerous ones” (van de Poel & Robaey 2017, p. 299). Although this principle can also be applied within the field of biotechnology, for instance, by using less hazardous organisms, there is a difference in that these principles are being applied to living organisms that can, therefore, act unpredictably (Robaey, 2018). In that sense, Robaey (2018) underlines that the first step in *doing* SbD in the field of biotechnology is to formulate strategies and measures beforehand (choice of organism, biocontainment, designing warning mechanisms) in order to be able to approach inherent safety.

## 4 RESULTS

Four themes were derived from the interviews and the stakeholder workshop. These themes help to clarify and structure the results in terms of differences in stakeholder perceptions of risks and safety, and expectations with regard to the concept of SbD. The identified themes are: (1) *risks and safety*, (2) *responsibility allocation*, (3) *inherent safety*, and (4) *the citizen's role*.

Section 4.1 provides an overview of the current regulatory setting according to the interviewees, and whether this corresponds to the state of affairs outlined in the literature. Each of Sections 4.2–4.5 provides a detailed overview per identified theme where any issues arose, and whether there were any contradictions between the interviewees’ statements3The interviews were held in Dutch, and the quotations from them have been translated into English. The original quotations can be requested from the corresponding author.. At the end of each section, the findings are linked to the concept of SbD. What implications this might have for future policymaking, as interpreted by the present researchers, are then discussed.

### 4.1 Current Situation

In the Netherlands, current legislative settings for biotechnology can be described as a *precautionary culture*, meaning that the Dutch government is held (end)responsible for inducing risks toward society, even unknown risks (Helsloot, Pieterman, & Hanekamp, 2010), assuming that research facilities or industry have complied with regulation. These regulations, which were developed in the mid 1990s, base their classification (GMO/non‐GMO) and the type of risk assessment needed on the process, rather than on the end product (as is done in the United States). A synthesis can derive exactly the same end product, but the path travelled—for example, via traditional mutagenesis or a synthetic pathway—is decisive for classification (GMO/non‐GMO).

According to all the interviewees, the current regulations for industrial biotechnologies in the Netherlands are unfit for future risk governance. In line with European legislation, the Dutch government uses the following definition of a GMO: “an organism with the exception of human beings in which the genetic material has been altered in a way that does not occur naturally by mating and/or natural recombination” (Ministerie van Infrastructuur en Milieu, 2014). This means that altered organisms that do not occur *naturally* need to be assessed on what risks they might pose to human health, animals and the environment, and fall under the Dutch GMO legislation. As techniques for genetic modification are developing rapidly and becoming increasingly more complex, basing the required type of risk assessment on the technology's process and its *naturalness* can become questionable. In that sense, we could argue that the definition of a GMO itself has become outdated.

This was acknowledged by several interviewees active in academia (AE) and policymaking (PM). For example, one of the interviewees (AE5) stressed that risk assessment in line with the current GMO regulation has become inadequate in terms of the technical details needed for proper risk assessment, that is, risk assessment tools for host organisms or vector list4Practical tools for risk assessment: identification of what risks accompany the use of certain host organisms or vectors. Offered by the Dutch GMO office (in Dutch: Bureau GGO). https://www.ggo-vergunningverlening.nl/ingeperkt-gebruik/hulpmiddelen-bij-de-risicobeoordeling/doorzoekbare-lijsten.. Thus, regulation no longer matches with what is being or is planned to be done in laboratory settings; it is lagging behind.

The end product can be the same, although different regulations may apply, which creates tension amongst researchers. Current governance is perceived as a burden by researchers. (PM1)

The standard vector list is no longer adequate; it is outdated. The researcher himself has been using vectors that they have tinkered with much more, so the list no longer matches [with reality]. (AE5)

The inadequate governance can be explained by the rapid developments in the field of biotechnology over the last decade. In particular, CRISPR applications have led to an increased pace in developments within this field. So far, the ruling of the European Court to classify CRISPR applications as GMOs has merely increased the complexity of handling such classifications, as such techniques do not match with the GMO classifications the GMO directive is based on. Although there was a consensus among the interviewees that current policy should be updated to conform with current biotechnologies, they also said that they expect that the rapid pace of these developments will continue, or perhaps even accelerate in the coming years. Interviewees from the domain of policymaking (PM) mentioned the SbD concept as a strategy to be able to anticipate future developments in industrial biotechnology responsibly, in addition to an update of current regulation. However, interviewee PM1 asks the question: “If SbD would become fully integrated, will policy become redundant?”.

### 4.2 Risks and Safety

The first identified theme revolves around perceptions of and the balance between risks and safety.

#### 4.2.1 How Safe is Safe Enough?

Regulation is an effective way to deal with technological risks. However, people from different contexts and different worldviews tend to have different perceptions of risks and safety (Adams, 1995, 2011; De Witt, Osseweijer, & Pierce, 2017; Hansson, 1989; Merad, 2020): what one person considers an unacceptable risk, another can find perfectly acceptable. Especially when dealing with uncertain risks, finding a balance in what level of risk is acceptable and coming to an agreement on this is very context dependent and can differ between stakeholders. Illustrative of this difference in perceptions are the responses from the interviewees representing academia (AE), industry (ID), and society (SO). The decision that industrial companies have to make about how “safe” they want their products to be is often based on the costs of “adding” safety to a product, and whether this addition outweighs another addition. “Where should the balance be between a safe product and an affordable one?” (ID1) Although it is hard to answer this question, when a product already complies with safety standards it is often a matter of the company drawing up a balance sheet. In academia, it is acknowledged that a technology can never be safe in an absolute sense: there will always be some risks that we have to accept. In that sense, some interviewees argue that current GMO regulation is too strict; too much emphasis within debates is put on risks while the risks are actually very small (AE1). The focus is too much on “safety on paper” (AE2).

Interviewees also pointed out that in societal debates, the emphasis is mostly on uncertainties that accompany a biotechnology, rather than the quantifiable risks. For the broader audience, accepting that a biotechnology can potentially harm the ones they love, directly or indirectly via the environment, is more complex due to people's values, and their perceptions of risks and safety, and of biotechnology in general (SO1). Emphasizing uncertain risks in the public debate might increase feelings of unsafety and lead to more reluctance to accept biotechnologies, thus hindering further development. In that sense, for society, determining what is not acceptable is easier than determining what would be acceptable. Interviewees from the field of policymaking acknowledged this difficulty in determining “what is safe” (PM3).

The extremes of something not being acceptable is easy. Within society, we are now in search of this level of acceptance [when something can be considered acceptable]. (PM3)

#### 4.2.2 How to Communicate About Safety?

Although the formal decision‐making process (i.e., licensing) on whether a technology is acceptably safe is based on legislation, peoples’ perception of what would be acceptable is also based on emotions, feelings, and personal experiences. Despite the complete absence of reported accidents in the field of white biotechnology in recent decades, some organizations still claim that biotechnology is an unsafe domain to operate in. These claims often rely on reported incidents or raised concerns coming from other strands of biotechnology, for example, gene drives (Scudellari, 2019) or germline editing (Rossant, 2018). Although these claims can sometimes be considered controversial, we can never guarantee that there will be no negative side effects in the long run, also for white biotechnology. Therefore, these organizations cannot be told that they are completely wrong. An interviewee active in the field of governance (PM3) addressed this when questioning whether we (the public) are actually concerned about safety itself or more about whether we feel safe, and to what extent this is influenced by the amount of discussion devoted to these topics.

Is it about safety or more about feelings of safety? These can be at odds with each other. For example, a fence around a prison can guarantee safety, while giving a sense of insecurity to local residents at the same time. Feelings of unsafety can sometimes increase more when more social debate is dedicated to it. (PM3)

A proposed solution to overcome this is to involve people more in the decision‐making process, thereby making them critically rethink the technology (SO1). A representative from industry (ID1) stressed that when a technology has undergone a sufficient risk assessment, it should be ready to be introduced into the market and within society. Elaborately informing people was not specifically mentioned by this interviewee, while all the other interviewees did mention this to a certain extent.

Other interviewees, however, pointed out the decreasing credibility of objective (scientific) information due to the increasing influence of industry within this domain (SO1), thereby questioning whether informing the general audience is effective. SO1 argued that, with an eye on SbD, industry influences the values associated with what would be acceptably safe. This raises a moral issue: “How critical do you have to be with regard to company interests within research?” (SO1).

#### 4.2.3 When to Consider Safety?

The interviewees held widely diverging perceptions on the acceptability of risks. In addition, another issue arises: when or where in the development process of biotechnology should safety aspects be considered? Although all interviewees acknowledged that safety aspects should be considered and acted upon during development, differences emerged in relation to emphasizing safety measures at the beginning or at the end of the development process. AE1 stressed that measures for safety should be taken into account throughout the process, thereby being adequately met by the end of the development process, namely when the technology enters the market stage. AE5 commented that the emphasis should be put on safety measures at the beginning of a biotechnology's development process, that is, during the design and idea phase. With regard to uncertain risks, this implies that the responsibility for determining what to identify as safety issues, what measures to take and what would be safe enough, would mostly be allocated to researchers. In contrast to AE5, AE1 specifically mentioned that you cannot expect only researchers to decide what would be safe enough. A different perspective was put forward by interviewee SO1, who argued that the negative or positive consequences of biotechnologies are often caused by people, not the technology itself. Although certain values may be embedded in a technology, this cannot guarantee that there will not be any misuse or different usage than originally intended. In that sense, SO1 emphasized that safety issues should mainly be addressed during the later stages of a biotechnology's development process, when a product is being introduced into society.

#### 4.2.4 Weighing Risks and Benefits During the Development Process

When dealing with uncertain risks, one way to determine what would be acceptable is to weigh the societal benefits of a technology against the known and unknown risks of a technology. Following this line of thought, PM2 argued that the specific moment at which risks are assessed during a technology's development process also calls for different standards. In that sense, benefits can be assigned a greater role depending on when risk assessment takes place, possibly creating a more appropriate balance between risks and benefits. For example, in the case of antibiotics, “the social benefit of this technology turned out to be huge” (AE4). However, a challenge would then be to determine what can and cannot be considered huge (PM1), and what *we* as a society “would be willing to give up for a certain matter” (AE1).

### 4.3 Responsibility Allocation

The second identified theme is allocating responsibility. When applying SbD as a way to anticipate uncertain risks, who should be accountable for the decision making on what is and what is not safe enough? Recalling the theoretical assumptions regarding SbD, a shared responsibility among stakeholders is desirable so that risks and safety aspects are fully taken into account throughout a technology's development process (Robaey, Spruit, & van de Poel, 2017; Stemerding & de Vriend, 2018; van de Poel & Robaey, 2017).

#### 4.3.1 An Equal Share?

The interviewees acknowledged that all stakeholders involved in the research, development, and further implementation of a biotechnology should have a shared responsibility for *being open*. Specifically, transparency in terms of raw materials, used products, processes, and techniques, and the subsequent risks and safety measures related to these. Although the interviewees agreed upon a shared responsibility, this does not mean that the weight of this responsibility should be equally divided. AE1 argued that, depending on the technical complexity of a biotechnology, researchers or stakeholders at the beginning of a biotechnology's development process should have a higher degree of responsibility. SO1 mentioned some concerns with regard to the “techno‐optimism” among researchers. Allocating higher degrees of responsibility to those considered experts in a technically complex matter (e.g., researchers) does not contribute to transparency in the decision‐making process on what would be acceptable in terms of safety and risks. In that sense, putting higher degrees of responsibility on these stakeholders would not necessarily lead to increased levels of safety, as societal concerns might be overlooked.

Everyone [should be held responsible]. But that is also dependent on what you're dealing with, how technically complicated that is, or the amount of expert knowledge necessary to make these decisions. (AE1)

#### 4.3.2 Allocating Responsibility

While some interviewees argued that researchers should have a higher degree of responsibility in terms of anticipating uncertain risks as they are situated at the “cradle” of a technology (AE1, AE5), others argued that, in this case, the Dutch government should be held responsible and take the lead in imposing regulation (AE4, PM1, PM3). In this way, the government functions as a controlling agent for researchers (AE4), thereby reflecting society's norms and values (democratic system). In addition, ID1 argued that industry should have a higher degree of responsibility, implying that also companies active in the field of industrial biotechnology should have the responsibility to be open about their products and processes. However, this might become problematic as not every company would want to go along with this level of openness for financial reasons or because of issues of confidentiality.

New developments create new uncertainties and therefore require reflection and new learning processes. Organising these processes around these new risk questions is where industry and scientists have a high degree of responsibility. But, for that, you will need an active government to stimulate it. (AE4)

You see, the industry naturally has responsibility for producing safe products. I think it would help if a company takes social responsibility into account and should therefore also provide information. (ID1)

**Fig 1:**
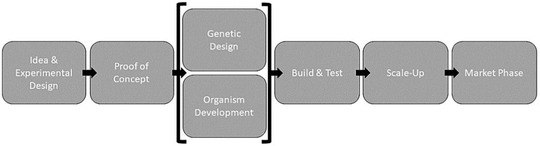
A simplified version of a biotechnology's development process. Phases with higher levels of responsibility would be upstream—the idea and proof of concept phase (e.g., researchers from both academia and industry), and downstream—the scale‐up and market phase (e.g., governmental regulation).

Graphically speaking, this means that higher degrees of responsibility are allocated to the beginning (idea and development phase) and the end (regulation and market implementation) of a biotechnological development process (Fig. 1).

### 4.4 Inherent Safety

As mentioned, strategies and measures for early and iterative safety considerations throughout a technology's development process are frequently referred to as *inherent safety*, even though a collective meaning of this notion is not shared by all stakeholders. Although all the interviewees acknowledged that technology can never be *absolutely* safe as the term *inherent* might suggest, their notions differed. Recalling the distinction we have made between product‐ and process‐applied SbD, inherent safety also conjures two different applications: one focusing on the product, the other on the process. This also leads to complexities in how we deal with risks; in a more quantitative way relying on known risks, or more subjectively relying on differing perceptions and values with regard to uncertain risks.

#### 4.4.1 Product Versus Process

SO1 argued that SbD is already being applied in industry, referring to the fact that their products should always be *safe*. This implies that, in this case, inherent safety is more associated with the product's technical aspects, and SbD is applied product‐wise. Within the domain of governance, inherent safety is considered a strategy to *do* SbD. In this sense, inherent safety is associated with process‐wise SbD, aiming to get as close as possible to the creation of *absolute* safety (PM3).

We have been working on Safe‐by‐Design for some time now. We define it more as a process. It is about taking safety into account as a value within a technological development. It is about thinking about safety during the development of products, which always includes safety. (PM3)

Although the distinction between these perceptions of inherent safety may seem a small one, it can have major consequences for how uncertain risks should be dealt with and for future risk governance. Interviewee AE3 mentioned that safety is always a dynamic, iterative process where multiple actors have to be taken into account, which is exactly where the challenge lies. “Applying the “helicopter perspective” to incorporate all stakeholders’ perspectives and opinions calls for prioritizing some over others” (AE3). However, AE3 also pointed out that scientists are under pressure to publish and therefore sometimes have to take risks. “Someone else will be ahead of you if you want to do it in a *safe* and *good* way” (AE3), illustrating the tension between the two mentioned notions: product and process applied. From a researchers’ perspective (product applied), prioritizing safety measures imposed by others (process applied) might result in safety measures that researchers may find excessive for the goal they have in mind. Finding a good balance in these prioritizations is crucial to ensure safety in a responsible way.

#### 4.4.2 Mismatches and Expectations

As an explanation for these differing perceptions of inherent safety, it is suggested that stakeholders simply have, or have access to different types of knowledge. From an academic's perspective (AE3, AE4, AE5), it was argued that policymakers can lack technological and/or scientific knowledge of the technologies for which they are preparing regulations. From a policymaker's perspective (PM1, PM2), it was argued that engineers might tend to focus too much on solely technical aspects, thereby overlooking unknown outcomes and uncertain risks that may occur later. However, policymakers putting pressure on researchers to take societal aspects into account is illustrative of the intertwining of the product and process application of SbD and inherent safety. This also raises the question whether researchers can, or should, consider safety issues arising from the process application, at the product application stage.

The interviewees argued that there are various reasons why researchers focus too much on the technology itself and too little on the societal implications. First, within industry, employees initially think of the interests of the company they are working for. “Their needs are simply put first” (ID1). Second, an incentive for researchers to actively think about “the unknown” is lacking (PM1). “What is in it for the researcher?” (AE5). Researchers are convinced that the technologies they are working with can be considered safe. “Tons of money coming from society is lost on unnecessary paperwork that does not necessarily contribute to safety” (AE2). This shows that for researchers, there is lack of understanding why emphasis should be put already at the beginning of the development process on safety issues that may not be relevant until later. Third, although risk assessment is currently also being done by researchers themselves, the preconditions have been formulated by others, namely policymakers. Because of this, the relevance or purpose of the conditions might not always be clear to them. In addition, PM3 argued that current regulations for risk assessment are not necessarily “risk‐based,” but more aimed at “legal risk assessment.” This lack of relevance originates, according to the interviewees, in academia, because researchers do not have a clear incentive to proactively think about future, uncertain risks. These examples strongly suggest that there is a mismatch between the perceptions of SbD and inherent safety between stakeholders, be it more technically or process applied.

#### 4.4.3 Expectations

We found indications that the term *inherent* can evoke expectations that might not be realized in practice. Especially for society, *inherent* safety could lead to high expectations of levels of safety. Although all the interviewees acknowledged these high or unrealistic expectations, a change in referring to this term differently has not been witnessed. For governmental institutions and policy bodies, the continued use of this specific term probably has some desired effects.

Either way, it has become clear that the perceptions and notions related to SbD are not aligned between stakeholders. In addition, the two applications of SbD and inherent safety (i.e., product and process) create tension between stakeholders at the beginning (researchers) and at the end of a biotechnology's development process (policymakers). This gives rise to the question of the extent to which SbD can act upon this by creating a dynamic, iterative environment in which stakeholders can communicate effectively. However, there is no clear agreement yet on how this could be established in practice. One step in the right direction would be to ensure that all stakeholders speak the same language.

### 4.5 The Citizen's Role

The last identified theme is the role of the citizen, namely the general public. Should the citizen have a role in the decision‐making process regarding the risks and safety measures related to industrial biotechnologies? One of the main questions that emerged from the interviews is whether the active involvement of citizens is often sought to push acceptance rather than to promote discussion. What is, or should be, the main reason to involve the public in these debates? And, more importantly, who should represent “the public”?

To start with, all the interviewees agreed that information regarding biotechnologies should be accessible to everyone who would like to be informed. However, opinions differed regarding the role that should be assigned to these people: the role of accepting or the role of choosing (SO1)? One interviewee from academia stressed that the only influence the general public should have on decision making is via the Netherlands’ democratically elected government (AE2). Another interviewee (AE1) applied a similar though slightly more nuanced perspective, arguing that it is the responsibility of parliament to express the citizens’ perspective one way or another, which can be done via debate, but also via the direct influence of citizens. However, both AE1 and AE5 stressed that the direct involvement of citizens is difficult as they cannot be considered experts in the field of biotechnology and would have difficulty understanding highly technical aspects. Thus, arriving at a consensus on the right balance between risks and benefits becomes complex. “A thorough background is needed to be able to correctly assess risks” (AE5). In addition, interviewee AE3 acknowledged that most citizens are not experts and therefore might have trouble indicating the right balance between risks and benefits. However, they would have a higher level of acceptance than “the professional” who had actually done the risk analysis and managed the process. In other words, it would be harder for citizens to accept as their threshold is higher.

Yes, vote! That is the only role [for the citizen]. We are a parliamentary democracy. (AE2)

A shared responsibility perhaps; citizen collectives and government. Reciprocity is essential to achieve safety. (PM1)

The citizen has certain values and thoughts, but these are often not included, or too late if they are included. Here it is assumed that the matters and discussions within biotechnology are too complex for the public. (SO1)

PM1, from the field of policymaking, argued that it is difficult to involve citizens in the decision‐making process regarding risks and safety, because they have very different perceptions of biotechnologies and risks. This difficulty in involving the citizen was also acknowledged by an interviewee from the societal domain (SO1), but was not stated as something that is impossible to achieve. SO1 stressed that the citizen has certain values and thoughts that should be taken into account in discussions revolving around biotechnology, but doubted whether and, if so, how these are included now. “It is often assumed that the subject matter is too complex for citizens anyway” (SO1). According to SO1, it is the role of policymakers to find out what these values of the public are and how to include these in policies. “Yes, the domain of biotechnology is complex, which makes it difficult but not impossible to have a broad discussion about this” (SO1).

No clear answer can be derived from the interviews and stakeholder workshop as to what the desirable role of the public should be within this debate. The interviewees’ opinions differed in terms of involving the public directly, or indirectly via representatives (e.g., the House of Representatives or the Senate—which comprise the bicameral legislature of the Netherlands, namely the States General). As including *everyone* would not be very practical, others who adhere to the second perspective argued that only the States General should be involved. This parliament is democratically elected by the public and has the means and desire to acquire the necessary knowledge and information to incorporate the citizens’ perceptions. Following that line of thought, the only true role for citizens would then be just to vote. However, when only the House of Representatives and the Senate are involved in such discussions, the public's trust (and access to knowledge) can become extremely important. Again, matters of trust are crucial here, as people vote for those they feel they can trust. Although there is no consensus on what exact role the public should have, we can say that within this debate the key should be facilitation, not pushing acceptance.

## 5 CONCLUSIONS AND FUTURE WORK

This study explored the different perceptions and associated notions of “risks,” “safety,” and “inherent safety,” and the implications of these for applying SbD as a governance instrument to anticipate uncertain risks. First of all, although SbD does show potential to deal with and anticipate uncertain risks that accompany emerging biotechnologies (e.g., CRISPR), the concept seems to create diverging expectations in terms of the aforementioned. Points of attention that arose from the conducted interviews and stakeholder workshop are the differences in the direct meaning and usage of SbD (i.e., process and product applied) and the notions created in relation to inherent safety by different stakeholder groups (science, policy, and society). Stakeholders that apply an SbD perspective product‐wise seem to put more emphasis on product specifications in terms of what would be safe enough, while stakeholders that apply SbD process‐wise put more emphasis on the process itself and the societal issues that accompany this process. This finding also applies to whether the public should be involved in these decision‐making processes, which makes more sense from a process‐applied perspective. These differences in applying SbD product‐ or process‐wise also lead to different judgments in terms of balancing risks, safety issues, and possible benefits, complicating collectively designing for safety. In addition, where this decision making should take place within a biotechnology's development process and who should be responsible for it remains unclear. There is a consensus that all stakeholders involved in this process should be responsible, but there is no agreement on whether the degrees of responsibility should also be equally divided, or whether some groups should bear greater responsibility than others.

Second, stakeholders’ expectations of SbD are not aligned. One way to resolve this issue would be to make others’ perceptions and expectations transparent to one another, thus enabling communication between stakeholder groups. However, more research is needed to establish whether there is indeed a lack of communication between these groups, and if so whether this relates to the two different applications of SbD and whether more transparency could solve this. But, most importantly, could SbD create an environment that enables this?

Third, in order to temper the high expectations that accompany the use of SbD and the term *inherent safety*, perhaps referring to, for example, Safer‐by‐design would be more appropriate in practice as this might create a more realistic idea of safety. However, it can be questioned whether this would solve the issue of the high expectations that accompany SbD and inherent safety, or whether the same problem would still exist, but then under a new name.

Finally, the concept of SbD is already being applied in other technical fields, namely, nanotechnology and chemical engineering. Future research could explore applications of SbD in these domains and investigate the extent to which these findings can be translated to the domain of industrial biotechnology, possibly contributing to define SbD within this context.

## 6 LIMITATIONS

We want to emphasize that all interviews were conducted within the Netherlands and can therefore only be associated with Dutch regulation concerning white biotechnologies (contained use). Although Dutch regulation is based on EU policy, we acknowledge that there are differences in regulation between EU member states. Also, although a broad range of stakeholders from the domain of biotechnology was interviewed, only a few of them have expert knowledge concerning risk governance within the EU. Therefore, findings from this study cannot be generalized and applied to regulation of biotechnologies in Europe or in general.
